# Editorial: Emerging multidrug-resistant bacterial pathogens “superbugs”: A rising public health threat

**DOI:** 10.3389/fmicb.2023.1135614

**Published:** 2023-02-01

**Authors:** Abdelazeem Algammal, Helal F. Hetta, Mahmoud Mabrok, Payam Behzadi

**Affiliations:** ^1^Department of Bacteriology, Immunology, and Mycology, Faculty of Veterinary Medicine, Suez Canal University, Ismailia, Egypt; ^2^Department of Medical Microbiology and Immunology, Faculty of Medicine, Assiut University, Assiut, Egypt; ^3^Department of Fish Diseases and Management, Faculty of Veterinary Medicine, Suez Canal University, Ismailia, Egypt; ^4^Department of Microbiology, Shahr-e-Qods Branch, Islamic Azad University, Tehran, Iran

**Keywords:** antibiotic resistance, multidrug-resistance (MDR), bacterial pathogens, superbugs, public health

The concern of antimicrobial resistance (AMR) is considered to feature among the top 10 threats to global health (World Health Organization, 2019, 2022). Several issues are recognized as relevant etiologic factors in the emergence of AMR, including worldwide misuse or overuse of antibiotics. According to recorded reports, low- and middle-income countries are pioneers in the promotion of AMR rates in comparison with high-income countries (Laxminarayan et al., 2016; Klein et al., 2018; World Health Organization, 2022).

Three major mechanisms relating to antimicrobial resistance, namely persistence, resistance, and tolerance, are detected among bacterial populations. According to published investigations, resistance increases the minimal inhibitory concentration (MIC) in populations of bacterial cells, while no increase can be seen in the MIC in cases of persistence and tolerance. Instead, an increase can be observed in the minimum duration for killing (MDK) among both 99% of tolerant (MKD99) and 99.99% of persistent (MDK99.99) bacterial cells within the relevant populations (Wistrand-Yuen et al., 2018; Balaban et al., 2019; Pacios et al., 2020).

The above adaptation mechanisms lead to deep global concern pertaining to current and classic pharmaceutical therapies used with different types of bacterial infectious diseases, e.g., urinary tract infections (UTIs). The appearance of biofilm formation in patients with chronic infectious diseases or recurrent infections is an outcome of the development of AMR among bacterial pathogens.

Due to the importance of the present topic, our team decided to present a relevant Research Topic in the journal Frontiers in Microbiology in order to direct particular attention to the issue and to gather a treasure trove of useful papers and sharp investigations. We attempted to address various points of view in the field in running the present Research Topic. In this regard, we succeeded in collecting and publishing six thematic and insightful papers from 60 authors from all around the world in our Research Topic. The collection presented in this Research Topic represents the latest data and information associated with the emerging multidrug-resistant bacterial pathogens, known as “superbugs,” which are known to represent a serious concern in the domain of rising global public health threats.

Lin et al. present a paper relating to the mobile (plasmid-mediated) colistin-resistance (*mcr*)*-1* gene in strains of *Escherichia coli* (*E. coli*) isolated from hospitalized companion animals in a veterinary (pet) hospital in Shanghai, China. Among 79 samples included in the study, taken from 22 cats and nine dogs, they detected seven *mcr-1* positive *E. coli* (MCREC) strains (8.9%) and 56 colistin-resistant strains (70.9%). Lin et al. demonstrated that all seven MCREC strains were multidrug-resistant (MDR) strains bearing a gene belonging to the type IV secretion system (T4SS). T4SS contributes to the horizontal transmission of antibiotic resistance and virulence factor genes through plasmids. This feature results in the accelerative dissemination of multidrug resistance among different populations of pathogens. Therefore, there is the possibility of the explosive dissemination of MCREC strains among human populations *via* companion animals.

In another paper associated with our Research Topic collection, Mazumder et al. investigated 17 non-lactose fermenting *E. coli* (NLFEC) bacterial strains that were isolated from clinical samples in Dhaka, Bangladesh. Specifically, eight strains of NLFEC bacterial cells were isolated from stool specimens and the remaining nine were isolated from urine samples. Mazumder et al. examined these in light of the global concern regarding the prevalence of clonal lineages at the international level. The prevalence of NLFEC strains was 10%; these involved the predominant sequence types (STs) of ST131, ST1193, ST12, ST73, and ST167, which were recognized as high-risk clones associated with epidemiological issues. Moreover, B2 was identified as the predominant phylogroup in the study by Mazumder et al. The results indicated that 63 and 33% of the MDR NLFEC strains were isolated from stool and urine samples, respectively.

Shafiq et al. present useful data and information regarding the first occurrence of the New Delhi Metallo-β-Lactamase (*bla*_NDM-5_) and tigecycline-resistance *tet*(X4) genes in the high-risk clone of ST648 associated with *E. coli*. This bacterial strain was isolated from a hospitalized patient (a 94-year-old man) with a UTI at Shantou Hospital, Guangdong, China. The isolated strain of ECCL209 encompassed several types of plasmids with the capacity for horizontal transmission of AMR genes. These plasmids caused the bacterial cells to function as a severely risky strain with the effective capability of disseminating AMR genes *via* horizontal transmission. Despite the low prevalence of this extensively drug-resistant (XDR) and MDR strain of ECCL209 among humans and animals, effective and rigorous monitoring is suggested to control this situation.

Ebrahim et al. demonstrated the antibacterial effects of several natural compounds extracted from red kidney bean (*Phaseolus vulgaris*) seeds against MDR strains of *Enterobacterales* members (e.g., *E. coli, Klebsiella pneumoniae, Salmonella typhimurium*, and *Proteus mirabilis*) isolated from 80 animals and 30 humans in the city of Fakous, Egypt. They showed that 97.92% of *Enterobacterales* isolates were MDR strains. In the study conducted by Ebrahim et al., the results obtained demonstrated that the red kidney bean protein composed of 7S and 11S globulin subunits has strong potential as a natural antibacterial compound that can be recruited against MDR strains of *Enterobacterales* members. Wang et al. present an effective methodology for control of plasmid replication in multidrug-tolerant (MDT) bacterial strains. The toxin–antitoxin system (TAS) can mediate and promote the growth of MDT and persister strains at the same time. In this regard, the authors selected *Pseudoalteromonas rubra* as an illustration of structural insights into the PrpTA toxin as an effective antitoxin system. Wang et al. present the assembly mechanism of the PrpTA (TAS) complex, its oligomerization process, and the related mechanism pertaining to the PrpTA TAS that controls the process of plasmid replication in *P. rubra*.

Finally, Lu et al. present an applicable real-time method for the detection of multiple resistance genes in food-borne pathogenic bacteria. In particular, they demonstrate an effective, rapid, and reliable detection method for monitoring and screening of antibiotic resistance genes in pathogenic strains of *Enterobacteriaceae*. Lu et al. employed recombinase polymerase amplification (RPA) assay in combination with a lateral flow dipstick (LFD) for simultaneous detection of *mcr-1*, *bla*_NDM − 1_, and *tet*(X4) genes. This combined detection method provides results within 40 min, with a limit of detection of 101 copies/μl.

## Author contributions

AA, HH, MM, and PB have contributed to the writing of the manuscript. All authors have read and agreed to the final version of the manuscript.
